# TGF-β1 polymorphisms -509 C>T and +915 G>C and risk of pancreatic cancer 

**Published:** 2017

**Authors:** Faegheh Behboudi Farahbakhsh, Ehsan Nazemalhosseini Mojarad, Pedram Azimzadeh, Faezeh Goudarzi, Amir Houshang Mohammad Alizadeh, Mehrdad Haghazali, Reza Mahmoudi Lamoki, Hamid Asadzadeh Aghdaei

**Affiliations:** 1 *Basic and Molecular Epidemiology of Gastrointestinal Disorders Research Center,Research Institute for Gastroenterology and Liver Diseases, Shahid Beheshti University of Medical Sciences, Tehran, Iran*; 2 *Gastroenterology and Liver Diseases Research Center,Research Institute for Gastroenterology and Liver Diseases, Shahid Beheshti University of Medical Sciences, Tehran, Iran*; 3 *Behbood Gastroenterology and Liver Diseases Research Center, Shahid Beheshti University of Medical Sciences, Tehran, Iran*; 4 *Proteomics Research Center, Shahid Beheshti University of Medical Sciences, Tehran, Iran*

## Abstract

**Background:**

Ninety percent of pancreatic cancer patients have less than 5-year overall survival and approximately 50% of cases were diagnosed with metastasis in the time of admission. Previous evidences have demonstrated the strong association between TGF-β1 variations and cancer susceptibility so far.

**Methods:**

A total of 78 patients with pancreatic cancer and 94 healthy controls were enrolled in this case- control study between 2007 and 2012. Genomic DNA was isolated from peripheral blood samples according to phenol chloroform extraction. The genotypes of TGF-β1 rs rs1800469 and rs1800471 were determined using the polymerase chain reaction-restriction fragment length polymorphism method.

**Results:**

The mean age of cases and the control group were 64.50 ± 13.718 and 40.12 ± 16.001, respectively. For polymorphism -509 C>T, the frequency of TT genotype were 31 (33.0), CT, 47(50) and CC, 16 (17) in control and 19 (24.4), 45 (57.7) and 14 (17.9) in cases respectively. In position +915 G>C, the frequency of GG genotype was 84 (89.4) and GC, 10 (10.6) in control and 71 (91.0) and 7 (9) in cases, respectively. We did not observe any significant differences in the genotype and allele frequencies of the TGF-β1-509 C>T (rs1800469) and codon +915 G>C (rs1800471) between the two study groups (P>0.05).

**Conclusion:**

we found that TGF-β1 gene polymorphisms rs1800469 and rs1800471 might not play a role in pancreatic cancer susceptibility in Iranian population.

## Introduction

Pancreatic cancer is the eighth most common cancer worldwide which is responsible for 25% of cancer-related deaths in western countries (1, 2). The overall 5-year survival rate with less than 5% indicates the fatal nature of this type of cancer. Smoking and diabetes have been appreciated as one of the main risk factors for pancreatic cancer progression (3, 4) approximately half of the patients were diagnosed with metastasis in the time of admission. Surgery is an appropriate approach in 15%-20% of patients and concomitant adjuvant chemotherapy might improve the 5-year survival rates up to 25% of patients (5, 6). The poor prognosis of pancreatic cancer requires extensive studies to shed light on mechanisms underlying the genetic and environmental risk factors related to its susceptibility. In the last few years, several studies have reported the association of pancreatic cancer susceptibility and variations in several cancer driver genes. Transforming growth factor beta 1 (TGF-β1) is a cytokine that belongs to transforming growth factor beta family and have both a tumor suppressor and pro-oncogenic activity in human cancers (7, 8). This multifunctional cytokine encodes a protein that plays a key role in differentiation, apoptosis, cell growth and immune cell function (9, 10). Involving in various cellular process including growth arrest and proliferation it is shown that it can improve the effect of anti-cancer therapy (7, 8). The overexpression of this gene has been demonstrated to mediate in angiogenesis, cell proliferation and migration (11, 12). In addition, single nucleotide polymorphisms (SNPs) are the most frequent genetic variations in the human genome. Several SNPs within genes have been reported to contribute to cancer susceptibility (13). Previous evidences have demonstrated the association between TGF-β1 variations and several diseases and malignancies so far (14-17). There are three common SNPs in the TGF-β1 gene including -509C/T (rs1800469), +869T/C (rs1800470), and +915G/C (rs1800471) which have been associated with susceptibility to several diseases and cancers (18-20). However, the role of SNPs -509C/T (rs1800469) and +915G/C (rs1800471) in gastric cancer predisposition have not been clarified yet. In the present study, we aimed to determine the association between TGF-β1 polymorphism, including -509 C>T (rs1800469) and +915 G>C (rs1800471) and cancer susceptibility in 78 patients with pancreatic cancer and 94 healthy controls in Iranian population.

## Patients and Methods

**Study Population**

A total of 78 patients with pancreatic cancer and 94 healthy control subjects without a family history of pancreatic cancer enrolled in this study. All patients had been referred to the Gastroenterology and Liver Diseases Research Center, Taleghani Hospital, Shahid Beheshti University of Medical Sciences, Tehran, Iran between 2007 and 2012. Written informed consent was taken from patients and the local ethics committee approved the study protocol.

**DNA Extraction**

Genomic DNA was isolated from peripheral blood samples according to the phenol chloroform extraction and ethanol precipitation protocol/method (21). The quality and standard quantity of the extracted DNA was then determined by NanoDrop 1000 Spectrophotometer (Thermo Fisher Scientific Inc; Waltham, MA). At the end samples were frozen at –20°C for further process.

**Determination of TGFβ genotypes**

The genotypes of TGF-β1, rs rs1800469 and rs1800471 were determined by polymerase chain reaction-restriction fragment length polymorphism (PCR-RFLP) method. Amplification of TGF-β1 fragments were performed in a 25-μl reaction mixture containing 2.5 μl of 10X buffer,0.5 μl of dNTP ,0.75 μl of Mgcl2, 0.5μl of each primer. The sequences of PCR primers and characteristics of restriction enzymes used for RFLP are summarized in Table 1. In addition 0.25μl (2.5unit) taq polymerase and 100 ng of genomic DNA was used to perform the PCR. the PCR condition for the SNP were as follows: 95 °C for 5 min; 35 cycles of 95 °C for 45s,62 °C for 35s,72 °C for 40s and a final extension at 72 °C for 10 min. Then RFLP procedure was performed with 15μl of PCR product and 0.5μl of restriction enzyme Eco81I for rs1800469 and BglI for rs1800471. Immediately PCR products were incubated at 65 °C overnight. The fragments were analyzed via 3% agarose gel electrophoresis. The green viewer staining was used to visualize the DNA band. The fragmented RFLP products are also presented in table1.

**Sequencing**

Sequencing method was done for 10% of the PCR products to confirm the RFLP procedure using an ABI PRISM 3130xL Genetic analyzer (Applied Biosystems®, Invitrogen Life Technologies, Carlsbad, Ca, USA) and the chain termination method.

**Statistical analysis**

Statistical analysis was performed with the help of the SPSS statistical analysis software package, version 13 (SPSS Inc, Chicago, IL). The statistical significance of the genotype and allele distributions between the case and control groups was determined by χ^2^ testing. *P *<.05 was considered statistically significant. All analyses were adjusted for possible confounder variables. Logistic regression was applied to calculate odds ratios (ORs) and 95% confidence intervals (CIs) for the association between each genotype and GC.

## Results

The characteristics of cases and control subjects enrolled in this study are presented in table 2. Totally 78 patients with pancreatic cancer and 94 controls participated in this study. The mean age of cases was 64.50 ± 13.718 and in the control group was 40.12 ± 16.001. In this study, we did not observe any significant differences between the cases and controls for the mean age or gender distribution which indicates that the matching according to these two variables was adequate. The size of product fragments in rs1800469 including CC genotypes was 117-36 bp, TT 153 bp and for CT 153-117-36 bp and for codon 25; +915 G>C (rs1800471), GG genotype was 252-212-60 bp, GC genotype 312-252-212 bp. The genotype distributions of this polymorphism among both groups were in Hardy–Weinberg equilibrium. In Table 3 the genotype and allele frequencies of the TGF-β1-509 C>T and 25 G>C (rs1800471) among the patients and control subjects are presented. For rs1800471 the frequencies of G and C alleles were 94.7% and 5.3% in controls and 95.5% and 4.5% in patient respectively. In codon -509 C>T, the genotype frequency for CC were 17.9% ,CT 57.7% ,CC 24.4% in cases and in codon 25 and GG genotype in case were 91.0% and for GC 9.0%. Based on our findings we did not observe significant differences in the genotype and allele frequencies of the TGF-β1 -509 C>T (rs1800469) and codon 25; +915 G>C (rs1800471) between the two study groups (P>0.05). Furthermore no significant differences between genotypes or alleles were found in case and control subjects. We also did not find a significant association between case and control groups when we stratified our study population according to gender Table 4.

## Discussion

Pancreatic cancer is a fatal disease with late diagnosis and lack of early symptoms, therefore, the majority of the cases identified at advanced stages. It has been demonstrated that the five-year survival of pancreatic cancer is < 5% (22). TGF-β superfamily play a significant role in the regulation of different cellular process, including tissue homeostasis, development, growth and regulation of the immune system (23). Association of gene polymorphisms and protein functions has been indicated in many studies so far. Several SNPs in TGF-β1 gene have shown to influence the gene expression and protein function (24-26). In the present study, we have evaluated the association of two polymorphisms including TGF-β1 -509 C>T (rs1800469) and codon +915 G>C (rs1800471) with pancreatic cancer risk in Iranian population. In the present study, in codon -509 C>T, the genotype frequency for CC were 24.4%, CT 57.7% ,CC 17.9% in cases and in codon +915: GG 89.4% and 10.6% for control and in patients were 91% and for GC 9.0%. According to our results, there were no significant differences in the genotype and allele frequencies of both TGF-β1 gene polymorphisms -509 C>T (rs1800469) and codon +915 G>C (rs1800471) between the two study groups (P>0.05). In line of our study, GUO-YANG WU, et al. study on 73 pancreatic head cancer patients revealed no association between TGF-β1-509 T/C Polymorphism and risk of cancer in German population (27). Another study by Penka N, et al. evaluated the association between 122 malignant melanoma (MM) cell lines and polymorphisms in cytokine genes including +915, +869, 509. They did not detect any association between +915, +869 polymorphisms and risk of MM. However, they found high expression of -509 TT genotype and decreased frequency of the -509 in MM patients (28). In meta-analysis by Yi Liu, et al. on colorectal cancer reported that TGF-β1 polymorphisms including +915 G>C (GG) and TGF-β1 −509 C>T (TT) were not associated with risk of colorectal adenoma and colorectal cancer (CRC). However, patients carry C allele of TGF-β1 −509 C>T had an increased risk of CRC (16). In consistent to our study, Armin Hosseini Razavi, et al. also did not find any association between TGF-β1 +915G/C and -509C/T gene polymorphisms and chronic hepatitis B in Iranian population (29). On the other study on Prostate, Lung, Colorectal, and Ovarian (PLCO) Cancer Screening Trial in 2007 Daehee Kang, et al. didn't observe any association between prostate cancer risk and TGF-β1 polymorphisms including 509 C>T and their haplotypic combinations in PLCO trial (30). In another study, Anna Liberek, et al. did not observe any significant correlation between 915G>C or 509C>T genotypes and plasma level, gene expression or clinical parameters in children with inflammatory bowel disease (17). Similar to our findings, Tamizifar, et al. also did not find any significant association between -509 C>T genotype and allele frequency in Iranian ulcerative colitis (UC) patients. However, they observed a significant difference in both allele and genotype frequency of -800G > A polymorphism of TGF-β1 and UC (31). LIU Dai-shun, et al. in 2010 also reported that TGF-β1 915G/C polymorphism was not associated with chronic obstructive pulmonary disease (COPD) susceptibility (32). It is interesting that the TGF-β have this ability to change its function from a tumor suppressor to an oncogenic status in the later stages of cancer development (33). In this study there were no statistically significant differences between cases and controls in terms of the frequent distribution of sex, history of diabetes, age, king and family history of pancreatic cancer (P>0.05). There were also no statistical differences between cases and control groups when we stratified the case and controls according to the gender status (Table 4). In the present study, we did not find any association between allele or genotype frequency of TGFβ polymorphisms including -509 C>T (rs1800469) and codon +915 G>C (rs1800471) and pancreatic cancer susceptibility. However, In Meta-analysis in 2012 by Yang Liu et al. on TGF-β1 -509 C>T polymorphism and cancer risk, shown that the latter polymorphism might have a protective role in colorectal cancer susceptibility and have a decreased risk of colorectal cancer, especially in Caucasians (34). Sunhong Hu et al., in 2012 revealed that the -509 T allele were significantly associated with reduced likelihood of Nasopharyngeal Carcinoma (NPC) susceptibility. They demonstrated that this polymorphism may play a protective role in susceptibility to NPC in Chinese population (35). In another study by Guan et al. on gastric patients shown that patients with TGF-β1 +915 CG rs1800471 and CC genotype had a poorer 2-year survival than patients with the GG genotype. However, they did not observe any association with TGF-β1 -509 C>T polymorphism and gastric cancer risk (36). The systematic review by Jian Min Zhang, et al. evaluated the association between 509 C>T polymorphism and digestive tract Cancer (DTC) risk. They demonstrated that 509 C>T polymorphism might be a modest risk factor for DTC, especially for Caucasians (20). In contrast to our findings Ren-Guang Tang, et al. study on esophageal squamous cell carcinoma cases reported that patients carrying GC/CC of rs1800471 polymorphism had an increased risk of ESCC and shorter overall survival. In their study they indicated that rs1800471 C allele increased the risk of ESCC in Zhuangese population, China (37). In other study by Xianglin Yuan, et al. evaluated the association of three SNPs in TGF-β1 gene with distant metastasis-free survival (DMFS) and overall survival (OS) in patients with non-small cell lung cancer (NSCLC) treated with definitive radiotherapy. They demonstrated that TGF-β1 -509 C>T polymorphism and rs1982073 could be useful biomarkers for predicting DMFS in patients with NSCLC treated with definitive radiation therapy. In their study they didn’t observe any association between genotypes of the TGF-β1 rs1800471 and DMFS or OS (38). In another study by Lei Zhang, et al. in 2012 reported that The frequency of TGF-β1 -509 T/C polymorphism was significantly associated with leakage of the biliodigestive anastomosis in Patients with Pancreatic Carcinoma (p=0.043). In addition, they didn’t observe any association between 509 T/C polymorphism and post-operative survival duration in related patients (39). In study by Smirne C, et al. on pancreatic adenocarcinoma patients revealed that TGF-beta1 serum  levels  were significantly higher in patients than control group. Their findings indicated that this putative gene might be a good marker for further pancreatic cancer therapeutic studies (40). In conclusion, according to our findings, TGF-β1 gene polymorphisms including -509 C>T (rs1800469) and +915 G>C (rs1800471) might not play a significant role in pancreatic cancer susceptibility in Iranian population. At the end, it is highly recommended to carry out large sample studies so as to elucidate the effect of these polymorphisms on pancreatic cancer susceptibility.

**Table 1:** Information for TGFβ1 SNPs rs1800471 and rs1800469 that included in this study

SNPs	Location	Primer Sequence	PCR Product Size	Restriction Enzyme	RFLP Fragment Size
rs1800471 (+915)	(G/C)	Forward Primer 5’-GTTATTTCCGTGGGATACTGAGAC-3’ Reverse Primer 5’-GACCTCCTTGGCGTAGTAGTCG-3’	524bp	BglI	G: 252+212+60 C: 312+212
rs1800469 (-509)	(C/T)	Forward Primer 5’-CAGTAAATGTATGGGGTCGCAG-3’ Reverse Primer 5’-GGTGTCAGTGGGAGGAGGG-3’	153bp	Eco81I	T: 153 C: 117 + 36

**Table 2:** Demographic characteristics of the study population

Variables	Controls (n=94)	Cases (n=78)	P-_value_
Age (Mean ± SD)^a^	40.12 ± 16.001	64.50 ± 13.718	<0.001
Gender, n (%)^b^			
Male	49 (52.1)	46 (59.0)	0.369
Female	45 (47.9)	32 (41.0)	

a According to The chi-square test results.

**Table 3:** A comparison of pancreatic cancer patients and healthy control samples of allele and genotype distribution of two SNPs included in this study

SNP	Variable	Male			Female		
		Control	Case	Adjusted* OR (95% CI), P-value	Control	Case	Adjusted* OR (95% CI), P-value
rs1800469 (-509)							
	Genotypes n (%)						
	TT	16 (32.7)	12 (26.1)	1.00 (Reference)	15 (33.3)	7 (21.9)	1.00 (Reference)
	CT	26 (53.1)	24 (52.2)	0.838 (0.247-2.842) 0.777	21 (46.7)	21 (65.6)	4.043 (0.847-19.286) 0.080
	CC	7 (14.3)	10 (21.7)	1.485 (0.296-7.455) 0.631	9 (20.0)	4 (12.5)	0.682 (0.110- 4.220) 0.680
	Alleles n (%)						
	T	92 (93.9)	89 (96.7)	1.00 (Reference)	86 (95.6)	60 (93.8)	1.00 (Reference)
	C	6 (6.1)	3 (3.3)	1.145 (0.538-2.437) 0.726	4 (4.4)	4 (6.3)	0.927 (0.404-2.126) 0.858
rs1800471 (+915)							
	Genotypes n (%)						
	GG	43 (87.8)	43 (93.5)	1.00 (Reference)	41 (91.1)	28 (87.5)	1.00 (Reference)
	GC	6 (12.2)	3 (6.5)	0.507 (0.088-2.916) 0.446	4 (8.9)	4 (12.5)	6.289 (0.699-56.583) 0.101
	Alleles n (%)						
	G	92 (93.9)	89 (96.7)	1.00 (Reference)	86 (95.6)	60 (93.8)	1.00 (Reference)
	C	6 (6.1)	3 (3.3)	0.526 (0.096-2.880) .0459	4 (4.4)	4 (6.3)	5.275 (0.713-39.033) 0.103

* Adjusted for confounder variables such as Age and Gender.

**Table 4:** Assortments analysis of gender group for two studied polymorphisms

SNP	Variable	Controls (n=94) n (%)	Cases (n=78) n (%)	Adjusted* OR (95% CI), P_value_
rs1800469 (-509)				
	Genotypes			
	TT	31 (33.0)	19 (24.4)	1.00 (Reference)
	CT	47 (50.0)	45 (57.7)	1.587 (0.632-3.984) 0.326
	CC	16 (17.0)	14 (17.9)	0.999 (0.312-3.197) 0.998
	Alleles			
	T	109 (58.0)	83 (53.2)	1.00 (Reference)
	C	79 (42.0)	73 (46.8)	1.046 (0.599-1.827) 0.874
rs1800471 (+915)				
	Genotypes			
	GG	84 (89.4)	71 (91.0)	1.00 (Reference)
	GC	10 (10.6)	7 (9.0)	1.418 (0.376-5.348) 0.606
	Alleles			
	G	178 (94.7)	149 (95.5)	1.00 (Reference)
	C	10 (5.3)	7 (4.5)	1.392 (0.383-5.051) 0.615

* Adjusted for Age as a confounder variable
